# Linking Soil Microbial Diversity to Modern Agriculture Practices: A Review

**DOI:** 10.3390/ijerph19053141

**Published:** 2022-03-07

**Authors:** Amrita Gupta, Udai B. Singh, Pramod K. Sahu, Surinder Paul, Adarsh Kumar, Deepti Malviya, Shailendra Singh, Pandiyan Kuppusamy, Prakash Singh, Diby Paul, Jai P. Rai, Harsh V. Singh, Madhab C. Manna, Theodore C. Crusberg, Arun Kumar, Anil K. Saxena

**Affiliations:** 1 Plant-Microbe Interaction and Rhizosphere Biology Lab, ICAR-National Bureau of Agriculturally Important Microorganisms, Maunath Bhanjan 275103, India; amritasoni90@gmail.com (A.G.); udaiars.nbaim@gmail.com (U.B.S.); pramod15589@gmail.com (P.K.S.); surinderpaulsandhu@gmail.com (S.P.); adarsh20149@gmail.com (A.K.); deeptimalviya77@gmail.com (D.M.); singh.shailendra512@gmail.com (S.S.); drharsh2006@rediffmail.com (H.V.S.); saxena461@yahoo.com (A.K.S.); 2 ICAR-Central Institute for Research on Cotton Technology, Ginning Training Centre, Nagpur 440023, India; pandiannkl@gmail.com; 3 Department of Plant Breeding and Genetics, Veer Kunwar Singh College of Agriculture, Bihar Agricultural University, Dumraon 802136, India; prakash201288@gmail.com; 4 Pilgram Marpeck School of Science, Technology, Engineering and Mathematics, Truett McConnel University, 100 Alumni Dr., Cleveland, GA 30528, USA; dpaul@truett.edu; 5 Department of Mycology and Plant Pathology, Institute of Agricultural Sciences, Banaras Hindu University, Varanasi 221005, India; 6 Soil Biology Division, ICAR-Indian Institute of Soil Science, Nabibagh, Berasia Road, Bhopal 462038, India; madhabcm@gmail.com; 7 Department of Biology and Biotechnology, Worcester Polytechnic Institute, Worcester, MA 01605, USA; crusberg@wpi.edu; 8 Department of Agronomy, Bihar Agricultural University, Sabour, Bhagalpur 813210, India; arunkumar20052@yahoo.co.in

**Keywords:** agricultural sustainability, soil microbial diversity, agriculture practices, tillage practices, microbial recruitment, pesticide effects

## Abstract

Agriculture is a multifarious interface between plants and associated microorganisms. In contemporary agriculture, emphasis is being given to environmentally friendly approaches, particularly in developing countries, to enhance sustainability of the system with the least negative effects on produce quality and quantity. Modern agricultural practices such as extensive tillage, the use of harmful agrochemicals, mono-cropping, etc. have been found to influence soil microbial community structure and soil sustainability. On the other hand, the question of feeding the ever-growing global population while ensuring system sustainability largely remains unanswered. Agriculturally important microorganisms are envisaged to play important roles in various measures to raise a healthy and remunerative crop, including integrated nutrient management, as well as disease and pest management to cut down agrochemicals without compromising the agricultural production. These beneficial microorganisms seem to have every potential to provide an alternative opportunity to overcome the ill effects of various components of traditional agriculture being practiced by and large. Despite an increased awareness of the importance of organically produced food, farmers in developing countries still tend to apply inorganic chemical fertilizers and toxic chemical pesticides beyond the recommended doses. Nutrient uptake enhancement, biocontrol of pests and diseases using microbial inoculants may replace/reduce agrochemicals in agricultural production system. The present review aims to examine and discuss the shift in microbial population structure due to current agricultural practices and focuses on the development of a sustainable agricultural system employing the tremendous untapped potential of the microbial world.

## 1. Introduction

Agriculture is the oldest profession of mankind, being practiced for centuries. To meet the ever-growing demand for food of the burgeoning human population at its present growth rate, the need to produce more food from ever-limiting resources is of genuine concern. Such a demand under the prevailing state of resources can only be met through increasing crop production with simultaneous decrease in the rate of diminution of available resources and utilizing them in a more sustainable manner. For example, the green revolution launched in India during the 1960s proposed yield enhancements through the use of agrochemicals, combining high-yielding (yet high nutrient-demanding) cultivars, the use of inorganic fertilizers to meet the crop nutrient demand. On account of these cultivars being sensitive to the onslaught of pathogens and insect pests, chemical pesticides have been used for plant protection, which resulted in an impressive increase in crop productivity [1,2] coupled with more or less a decrease in the quality of associated natural resources. The total outcome was greater than expected, and the country of India, once hunger-stricken, became a food grain exporter. The fear and pain of hunger was so deep that in the propensity to increase yields we, as a society, ignored the ill effects of many practices in the package of the green revolution on soil and human health. As a result, most of the natural resources, i.e., soil, water, and the environment itself, became polluted with agrochemicals owing to their excessive and injudicious application. The lack of local soil testing facilities led to blanket recommendations for crop nutrition in a blind race to increase yields. Inorganic fertilizers were used in amounts in excess to what was really needed. The application of agrochemicals, particularly chemical pesticides, for plant protection posed numerous threats to human health and life as whole. Pesticide residues have great impacts not only on the site of their application but also have reached environments far from where they were used [3]. This issue is especially of concern in developing countries, where large populations live in close proximity to farm land, often leading to direct exposure and causes severe health issues in humans [4]. Several improved agricultural technologies have been adopted for enhancing productivity without taking the sustainability of the system into account. Now that the ill-effects of injudicious use of agrochemicals have become rampant, we have begun to realize the need of ensuring long-term sustainability with optimal resource use and without negative effects in the restricted land available for agricultural cultivation. This has led to the emergence of a variety of beneficial management practices [5]. The availability and productivity of agricultural resources including water, energy, and land varies enormously between regions and production systems, and competition for efficient and judicious use of these resources will further intensify. Application and modification of different agricultural practices coupled with high inputs of agrochemicals can alter the soil microbial communities in general and functional communities in particular.

There is urgent need for ecologically sound agricultural advancement, which apart from being eco-friendly should also enable us to feed the ever-increasing population against the backdrop of ever-changing climatic conditions. It is evident from the fact that conservation of the environment and natural resources has become important considerations in agriculture today. In traditional agriculture, more tillage is preferred for better harvest, but in modern agriculture, no tillage or zero tillage with better residue management is preferred, as this seems to be one of the most important factors for long-term sustainability of agricultural ecosystem. Conservation agriculture-based farm practices advocate use of organic inputs and minimal tillage, whereas traditional production systems lay emphasis on maximizing yields using inorganic fertilizers and chemical pesticides with enhanced tillage. Such practices have led to deterioration of biological and physicochemical properties of soil and associated ecological systems.

With modernization of civilization, different developmental activities have brought much change in land utilization. Different anthropological activities have obligated changes in agricultural practices, which have led to shifts in soil microbial community structure altering physicobiochemical properties of soil [6]. Commonly used organic and inorganic agricultural inputs, following agricultural interventions, have a significant impact on soil microflora [7] and have a great role in determining the microbial community structure in the soil. It has been recognized that tillage may alter the physical structure, moisture, soil temperature, aeration, and rate of crop residue degradation [8] and decreases soil macroaggregation [9]. Minimal soil disturbance through adaptation of zero tillage or no till practices causing minimum soil disturbance is, therefore, preferred over conventional tillage in order to maintain sustainable soil health and crop productivity. The success of crop rotation depends largely on efficient recycling of nutrients, which is chiefly controlled by microorganisms and enzymes produced by them in the soil environment [10]. A rather high microbial activity is found in the surface layer of soils under no tillage systems with crop residue mulch as compared to conventional tillage without mulching.

Monoculture system of agriculture may change soil parameters, particularly species richness, microbial activity, and community structure. Changes in land use patterns have been reported to significantly affect the microbial population dynamics in soil [11]. Along with tillage, poor residue management and non-scientific cultivation practices in this regard have their own impacts, not only on soil biological activities, but also on the whole process of residue degradation [12]. The influence of agricultural residues on the microbial diversity of soil is well documented [13]. Crop diversification, however, is being recommended and to a great extent is being used for enhancing land productivity. Beyond a certain point, crop diversification also influences microbial diversity. Different plants release varied root exudates, a wide range of compounds, which, in turn, determine the microbial community structure [14]. Furthermore, crop diversification leads to diversity in carbon-rich compounds present in the rhizosphere, which ultimately influence the diversity of native microbial species. The fact that stressed or challenged plants secrete a number of molecules which act as signaling compounds between plants and microorganisms also becomes relevant in this respect. Soil microorganisms are known to use these chemical-based messages in order to communicate with plants, and by sensing these molecules, they contribute to activate defense mechanisms in the plant under stressed conditions [15].

In view of the above, there is an urgent need to understand and quantify the impact of different agricultural practices on soil microbial communities in general, and on functional groups in particular. Microorganisms are well-known and useful in reducing some concerns associated with chemical fertilizer and pesticide applications [16]. The purpose of this review is to provide an overview and in-depth understanding of the impact of different agricultural production technologies, including the application of inorganic inputs and cropping system on changes in soil microbial diversity. This information may be utilized in developing package of practices and shaping of agricultural policies for greater benefits of the farming community as well as other stakeholders.

## 2. Microbial Recruitment in Different Tillage Practices

Agricultural management practices that promote soil organic matter (SOM) accumulation and retention enhance bacterial biodiversity. Tillage has been found to impact soil bacterial diversity negatively, but it did not affect arbuscular mycorhizal fungi (AMF), fungal, or functional diversity. However, organic farming did not affect soil biodiversity as compared to conventional farming [17]. Both species loss and changes in the relative abundance of species present can affect ecosystem functioning and subsequent ecosystem services [18]. Wolińska et al. [13] observed a higher number (65) of dominant bacteria operational taxonomic units (OTUs) in non-cultivated soils than that in cultivated soils (47). Li et al. [19] reported that a microbial community of *Camellia oleifera* forest changes significantly due to different agrofarming activities. Furthermore, Li et al. [19] have also observed that bacterial community composition, species richness and the fungal community significantly differed under different management practices, whereas fungal species richness remained unaffected [20]. The highest fungal richness was obtained under cover crop. Shifts in microbial community structure and the abundance of various plant-beneficial and detrimental soil microorganisms have been shown to influence the productivity and stability of the agroecosystems [21].

Tillage has been recognized as the most important driving factor in influencing soil microbial community in general and bacterial diversity in particular [22]. Different tillage practices are known to influence soil organic carbon, moisture, and physical properties by and large. Since soil enzyme activity is greatly influenced by these parameters, the type of tillage practice has direct impact on soil enzyme activities [23]. Intensive tillage and application of chemical inputs in higher rates have widely been exploited in conventional agriculture with an aim to increase production to meet the growing food demands of an ever-increasing human population. As a result, soil binding capacity decreased to considerable extents and agriculture fields became more prone to erosion by surface runoff, which led to non-point sources of pollution across the world [24]. The resultant pollutants including plant nutrients, organic matter, chemical pesticides and soil sediments are transported through precipitation and irrigation from field to surface water. About one third of these eroded pollutants flow into rivers and lakes and in addition to pollution of water bodies, leading to a reduction in volume of top soil and the amount of plant nutrients applied [25,26]. Amidst increased concern over soil quality and health, an alternate method termed “conservation agriculture” has gained greater attention in preserving physicochemical and biological properties of soil [27]. This approach maintains soil stability to effectively manage surface runoff and minimize the possibilities of pollution as mentioned above. Conservation agricultural practices such as no-tillage (NT), also referred to as zero-tillage, and organic farming have improved soil health, namely, soil microbial diversity in general and soil microbial community stability in particular [28].

### 2.1. Impact of Conventional Tillage vs. No-Tillage on Soil

Conventional tillage (CT) including disc plough, moldboard plough, and chisel plough, have resulted in severe land degradation and have posed risks to the concept of sustainable agriculture [29]. CT practices resulted in disruption of soil aggregates, compactness of soil, and reduction in spaces between soil particles, which led to alteration in movements of water and gas into the soil, and thus, eventually affect the soil as a habitat for living organisms and microbial functional diversity [30,31]. NT practices can minimize such soil disruption and soil organic C oxidation while enhancing the soil C content, soil aggregation, and rate of infiltration of water [32] into it. It results in decreased detachment of soil particles, thereby decreasing both soil erosion and transport of pollutants [33]. It was reported that about 11% of arable land has adopted no-tillage practices globally and this has reduced the runoff by 64.9% [34]. NT practices result in a greater content of soil organic matter, nutrients, and a minimal oxidizing environment with the absence of disruption of soil layers, which ultimately helps in stabilizing the extracellular enzyme pool [31]. NT is a more sustainable practice for improving soil health and microbial diversity [23]. Irrespective of nutrient applications, NT practices have shown an increase in the soil enzymatic activity as compared to CT, which may be attributed to an increase in dehydrogenase and urease activities [35]. Furthermore, NT treatments showed significant increases in extractable soil nutrients, such as calcium (Ca) and magnesium (Mg), than conventional tillage [36], which might directly/indirectly influence the structural and functional community of soil microorganisms.

### 2.2. Soil Microbial Diversity in Different Tillage Practices

Minimum tillage (MT) in combination with organic farming seems to be an effective strategy to enhance soil microbial biomass and abundance [37]. The microbial community structure shifts towards bacteria under organic farming, as bacteria respond more strongly to agricultural practices than other microbial groups [38]. NT in combination with organic farming enhanced soil microbial properties more than either of individual practice and CT [28]. NT together with cover crops increased substrate diversity and thereby influence the soil microbial enzymes production [6,35]. This is because of greater utilization of carbohydrates in the top soil (0–10 cm), which contains rich organic matter and sugars released from decomposition of agricultural residues. Furthermore, carbohydrates have also been found to maintain and stimulate the soil microbial activity in rhizosphere as compared to non-rhizosphere soil [39].

Sun et al. [37] evaluated the effect of long-term practice of organic farming and MT on microbial community structure and microbial diversity. The results showed a positive influence on soil microbial biomass, total phospho-lipid fatty acids (PLFA), Gram-positive bacteria, Gram-negative bacteria, and mycorrhiza. The increase in the Gram-positive bacterial population was reflected by an increased content of bacterial muramic acid. MT had significantly increased microbial biomass N and fungal PLFA. Nivelle et al. studied the combined effect of NT with cover crops under N-fertilization over a period of five years in cereals (wheat and corn) and legume (pea and flax) crops rotation influence the microbial activity in the soil [35]. It increased the total nitrogen and total organic carbon coupled with an increased soil microbial functional activity and their diversity when a cover crop was associated with NT. CT, on the other hand, showed a negative impact on soil C and soil N availability and enzyme activity. The incorporation of only wheat straw resulted in high C:N ratio, which was not compensated in conventional tillage [35]. An increase in soil C and N by 19% and 10% was observed, respectively, under minimum tillage with N-fixing cover crop systems than with conventional tillage without using any cover crop [36]. Most of the studies assessing impact of tillage on microbial community were carried out focusing on (a) soil properties (physicochemical and biological) under different tillage practices, (b) no-tillage (NT) with an eye on increase in microbial diversity and richness, and (c) significance of relationship between soil C and N with microbial community composition [23,35]. Moreover, long-term application of NT resulted in significant increase in unique OTUs, species richness, and evenness and higher Shannon index, while lower Simpson index was observed as compared to CT. This is attributed to greater availability of substrate due to higher soil organic C as food, which ultimately increased the bacterial diversity [23]. Legrand et al. [40] observed 1822 OTUs representing 85 genera under CT while 1720 OTUs representing 105 genera under MT, which indicated the increase in species richness despite less OTUs under MT. They also reported a lower α-diversity with higher β-diversity in soil under CT than MT. CT negatively affected the soil fertility or soil nutrient availability which resulted in reduced microbial diversity [40]. In contrast, some recent studies reported a higher microbial diversity in conventional tillage [41,42]. However, functional microbial diversity was always higher under NT or MT practices when compared to CT. These discrepancies may be attributed to variations in carbon and nitrogen inputs, soil pH, and temperature [35]. Furthermore, higher diversity and biomass of soil microorganisms, such as bacteria (both Gram +ve and Gram –ve), fungi, and actinomycetes, were observed in no-tillage systems compared with conventional tillage systems [28].

#### 2.2.1. Bacterial Diversity

*Proteobacteria* (α, β and γ), *Actinobacteria*, *Bacteroidetes*, *Acidobacteria*, and *Chloroflexi* are the five major phyla representing more than 80% of the bacterial diversity under both NT and CT systems in a winter wheat crop. NT practices harbored relatively higher abundance of *Proteobacteria*, *Actinobacteria*, and *Bacteroidetes* and lower abundance of *Acidobacteria* than CT. At genera level, *Arthrobacter* and *Streptomyces* were predominant in both systems. However, NT had a higher abundance of *Sphingomonas* and *Pseudomonas* while CT had a higher abundance of *Acidobacteria* and *Chloroflexi*. The predominance of *Streptomyces* (*Actinobacteria*) in both NT and CT may be due to their ability to produce spores, which might have helped them to survive under both conditions [23]. The predominance of *Bacteroidetes* under NT is attributed to their ability to rapidly utilize bio-available organic matter and copiotrophic characteristics, which occur in soils with high carbon availability [23,43]. No significant difference in *Arthrobacter* was observed in either system. This suggested that *Arthrobacter* was an oligotroph and is able to degrade extremely recalcitrant substrates with slow growth [23]. A lower abundance of *Acidobacterium* in an NT system compared with a CT system suggests their wider tolerance to nutrient-poor habitats [44] and is also indicative of the fact that external disturbances did not significantly affect them [23].

#### 2.2.2. Fungal Diversity

The fungal population is expected to be higher under minimal tillage (MT) or no-tillage (NT) conditions due to minimal disruption of the hyphal network of fungi and greater resistance to degradation of their chitinonus cell wall [45,46]. The higher ratio of fungi to bacteria indicated the stable ecosystem in NT soil, which was more similar to an undisturbed soil microbial community. The fungal hyphal network can be better established under an NT system as compared to the CT system as the mechanical disturbance is greater in the CT system. These hyphal networks can effectively translocate nutrients to the plants [45]. Fungi also have an added advantage of adaptation to cooler and moist environments prevalent in NT systems [45]. Some reports, however, indicate deviation from this fact [36,45]. FAME analysis revealed that Actinomycetes and Mycorrhizal fungi were abundant in NT treatments, while saprophytic fungi were abundant in CT treatments [36]. Mycorrhizal fungi play a vital role in protecting soil organic carbon by facilitating formation of macro-aggregates and its stabilization in addition to mobilization of nutrients. The effects of these mycorrhizal fungi could be affected due to interruption of their hyphal network during tillage [47]. Mycorrhizal fungi are enriched under NT conditions in most cropping systems [28,36].

### 2.3. Other Properties

The extent of soil disturbances can be measured by different indicators, which include microbial, enzymatic, and metabolic activities. The increased activity of soil enzymes such as β-glucosidase, β-glucoaminidase, and phosphodiesterase under NT treatments was attributed to greater accumulation of plant residues over many years [48]. Soil microbial diversity and community are closely associated with quality and quantity of soil nutrients and soil C content. Therefore, these parameters may act as a sensitive indicator to predict the prevalence of the soil biological community [49]. Generally, soil microbial biomass (SMB) is used as an important parameter for determining soil quality and is expected to be greater under MT or NT in most of the cropping systems [50]. However, it is not always the same and sometimes no significant changes in SMB are observed, which suggests that SMB alone may not be a good indicator in determining soil quality, particularly in low residue cropping systems such as cotton [36].

Fluorescein diacetate (FDA) is predominantly used as an indicator for soil health since it can be hydrolyzed by non-specific esterases, proteases, and lipases. Rincon-Florez et al. [51] evaluated the impact of occasional strategic tillage (ST) on microbial communities using two tillage systems and two stubble management practices. They reported that no significant effect of ST on biological attributes was observed except for total enzymatic activity. The enzymatic activity was increased significantly in CT-SR (conventional tillage-stubble retention) as compared to NT-SR. This difference may be characterized by a significant increase in the bulk density under CT-SR treatments. The absence of changes in microbial diversity may be related to high resistance and/or resilience of soil microbial communities. The results of PCA analysis with the study in which two factors tillage (tillage vs. no-tillage) and fertilizer input (chemical vs. organic) were taken, which clearly indicate the divergence in microbial community due to long-term following of different agricultural practices [28].

Higher P, Ca, and Mg were observed under NT treatments which may be attributed to perpetual increase of soil organic matter as it acts as a source of nutrients through mineralization and releases organic acids which chelate available nutrients in soil. Levels of these nutritional elements decreased with increased N, which also results in decreased soil pH. Long-term application of N-based fertilizers such as ammonium nitrate resulted in increased acid production by ammonia oxidizing bacteria. This led to increased soil acidity, which, in turn, reduced the availability of other nutrients, such as P, Ca, and Mg [36].

Despite the undisputed role played by soil microbial communities in maintaining soil health and enhancing crop productivity, an understanding of their response to long-term agricultural practices is still limited [28]. The advent of high throughput sequencing helped us to detect even less abundant microbes, which was not possible earlier [52]. Still, the response of soil microorganisms to different farming practices is poorly understood [35]. An extensive study on impacts of various tillage practices [23] and inputs (chemical and organic) on microbial communities is needed if we are to attain a decent level of sustainability in soil health and in agriculture overall.

## 3. Microbial Recruitment Affected by Crop Cultivar Rhizosphere

The plant rhizosphere is inhabited by the highly diverse microbiota and can hold up to 1×10^11^ microbial cells per gram of plant root [53]. More than 30,000 prokaryotic species have been reported so far [54], which indicates the magnitude of microbial diversity. The collective genome (the rhizosphere microbiome) of this diverse microbial population is very large in comparison to the host plant genome and is supposed to play a very crucial role in the host survival. Thus, being crucial for plant health, it is also expressed as the plant’s second genome [55]. The plant rhizosphere associated microbial communities also plays a central role in carbon sequestration, proper functioning of ecosystem as a whole, and regulation of nutrient cycling in natural as well as agricultural and forest ecosystems [56]. Diversity of microbes inhabiting the plant rhizosphere and their complex interactions with the host plant significantly affect plant morphology, physiology, plant growth, development, and health [57]. Each plant has various biochemical processes ongoing, which culminate in specific micro-environmental conditions in the rhizosphere that seem to provide a dwelling ground for the specific microbial population subset with distinct functional capabilities [58]. Any factor that changes the microbial community structure, composition, or its activities has marked effects on the normal growth and development of the plant in a particular environment. Thus, in order to understand the composition of microbial community structure in the rhizosphere of a particular plant and its complex plant–microbe interactions, it is very important to explore the various environmental and physiological factors which play crucial roles in this complex and dynamic process.

### 3.1. Factors Affecting Rhizosphere Microbial Population

#### 3.1.1. Soil Type

Many factors, namely the soil physiochemical profile, the environment as well as the type and developmental stage of the particular crop/cultivar, formed a specific niche with the unique micro-environment, and altogether play important roles in shaping and determining the microbial community structure and composition in the rhizosphere of the plant [59,60]. As soil is the ultimate source of all the nutrients needed for the development of a plant, the soil type, its chemical and physical composition, as well as nutrient profile has a huge effect on the plant physiological process [61,62]. İnceoğlu et al. [63] proposed and confirmed that soil type plays the most significant role in determining the structural and functional community structure of the potato rhizosphere-associated bacteria. İnceoğlu et al. [63] also confirmed that the same potato cultivars grown in two different soils had different rhizosphere inhabiting microbes with different functional capabilities. Breidenbach et al. [64] also studied the dynamics of rhizospheric microbiota of rice plants and further confirmed that community structure is greatly affected by the specific soil type and the environment (i.e., rhizosphere versus bulk soil) than did time (e.g., plant growth stage).

#### 3.1.2. Crop Cultivar

Researchers have shown that different plant species growing in the same soil type can have a totally different rhizosphere-associated microbial population structure [65,66,67]. However, some plant species can recruit similar microbiota even in different soils [68]. Reports also demonstrated that even within species, different genotypes can have distinct rhizosphere microbial communities [69]. All these reports further suggest that the host plant plays a very crucial role in shaping microbial community structure associated with its rhizosphere. Researchers have proven that the rhizosphere-associated microbial population composition is also dependent on the host plant genotype (cultivar) [70]. This is termed the “rhizosphere effect”, which describes that the root-associated microbiota community structure often remarkably varies not only across host plant species but also among different genotypes within a single species [55,71,72]. Jiang et al. [73] revealed that blueberry host cultivars exerted substantial effects on the root-associated bacterial diversity along with complex co-occurrence networks and that host genotype directly influenced the microbiota profiles.

#### 3.1.3. Composition of Root Exudates

Root exudates of plants are known to consist of compounds acting as attractants for the specific microbial community to which these exudates provide nutrition, and thus, may play key roles in the determination of microbial population dynamics in the rhizosphere of the plant [74]. The active root secretions or root exudates comprise a diverse range of low molecular weight compounds released by the host plant which enable it to modulate (stimulate or suppress) the growth and colonization of selected species of rhizosphere-associated microbes [75]. The root exudates are composed of various ions, enzymes, free oxygen and water, mucilage, and a diverse set of primary and secondary metabolites, which are utilized by the microbes as a source of carbon [76,77]. Furthermore, root exudates can broadly be divided into two classes of compounds: (a) a low molecular weight fraction which is highly diverse and is composed of amino acids, organic acids, sugars, phenolics, and other secondary metabolites, and (b) a second class composed mainly of mucilage (polysaccharides) and proteins, but in a less diverse high molecular weight fraction [77]. Some root exudates also contain chelating agents which form complexes with metallic micronutrients including iron, zinc, manganese, and copper and, thus, affect the nutrient availability in rhizosphere soil [78].

The amount and composition of the root exudates has also been found to be affected by nutrient availability, soil type, physiology, growth, and developmental stage of the plant [79]. The root exudates from plants in certain sets of conditions can favor the establishment of a distinct rhizosphere microbial community by providing wide yet specific varieties of carbon sources [57]. Root exudate components such as carbohydrates and amino acids act as stimulants and help plant growth promoting bacteria (PGPB) colonization through chemotaxis [80], a well-known mechanism for the establishment of interactions between soil microbiota and host plants within the rhizosphere [81]. Weert et al. [82] reported the chemotactic effect of root exudate components on the flagella driven motility of *Pseudomonas fluorescens* and elucidated its role in tomato root colonization. Flagella driven motility in microbes is considered an important trait which can significantly affect the population structure of competitive pathogens and beneficial microbes in the plant rhizosphere and thus facilitate various microbe–microbe and plant–microbe interactions [83]. Early host recognition by bacteria is also mediated by the bacterial Major Outer Membrane Protein (MOMP). *Azospirillum brasilense* MOMP exhibiting stronger adhesion to membrane-immobilized root extracts of cereals as compared to legumes and tomato extracts, one example which suggests that MOMP may act as an adhesion factor, playing a key role in bacteria-to-root adsorption and cell aggregation by the bacteria allowing colonization within a specific host plant rhizosphere [84].

Root exudates are known to influence and maintain rhizosphere-associated core and cultivar-specific microbiota [73]. Secondary metabolites representing the specific subclass of flavonoids are known to play an important role in the very specific plant–microbe interactions between legumes and nitrogen fixing rhizobacteria. These interactions further enable a specific strain of rhizobacteria to form nodules within cells of its specific leguminous plant host [85,86]. Peters et al. [85] established that isoflavonoids are specifically produced only by leguminous plants and they are known to regulate the expression of nod genes in specific nitrogen fixing microbes. Apparently, flavonoids are perceived as aglycones by the rhizobacteria, and interact with the nodD protein (a LysR-type regulator) and alter its conformation to facilitate binding to nod box elements in the promoter regions of the nod genes, inducing expression of nod genes to synthesize Nod factor molecules [86]. Chemically, Nod factors are lipochitooligosaccharides, usually consisting of four or five β-1,4 N-acetylglucosamines, with the terminal nonreducing sugar N-acylated by a 16–18 carbon fatty acid. Nod factors may also contain acetate, sulfate, or carbamoyl groups, or different sugars, such as arabinose, fructose, and substituted fructose. All these chemical modifications form the basis of host specific recognition of a specific nod factor in legumes. For instance, daidzein and genistein, isoflavonoids produced by soybean (*Glycine max*), positively regulate nod gene expression in *Bradyrhizobium japonicum*, but negatively regulate nod gene expression in *Sinorhizobium meliloti*. The nod gene expression in *S. meliloti* is instead found to be specifically induced by luteolin [85].

In plant–mycorrhiza interactions, signaling molecules known as ‘branch-inducing factors’ present in the root exudates of plants critically help mycorrhizal fungi in hyphal branching, root colonization, and in establishing a symbiotic relationship with the host [87,88,89]. Akiyama et al. [90] have isolated a ‘branch-inducing factor’ chemically identified as 5-deoxy-strigol, a strigolactone, from the root exudates of *Lotus japonicus*, which at very low concentrations induced extensive hyphal branching in germinating spores of the arbuscular mycorrhizal fungus, *Gigaspora margarita*. Nutrient availability to plant hosts has also been reported to affect the production and/or exudation of ‘branch-inducing factor’ in its root exudates. Nagahashi and Douds [91] reported that root exudates from plants growing in phosphate (P)-limited conditions had high activity of branching factor compared with plants growing in phosphate (P)-sufficient conditions.

Secondary metabolites in plant root secretions also inhibit the growth of particular microbes [92] and, thus, influence the microbial population dynamics in the rhizosphere. Bais et al. [93] reported the secretion of rosmarinic acid in the hairy root cultures of *Ocimum basilicum* and its role in exhibiting specific antimicrobial activities. A benzoxazinoid, 2,4-dihydroxy7-methoxy-2H-1,4-benzoxazin-3(4H)-one (DIMBOA), present in large quantities in *Zea mays* root exudates is reported to exhibit potential antimicrobial activity as well as to act as selective chemotactical attractant for the plant beneficial rhizobacterium, *Pseudomonas putida* KT2440 [94]. Plant secondary metabolites are also reported to interfere positively or negatively with ‘quorum sensing’ (QS)-regulated responses by altering the expression of several QS-related genes in bacteria. As QS is very important for cell-to-cell communication and colonization in bacteria, these metabolites may influence the population structure of microflora in the rhizosphere. Several compounds interfering with plant-bacterial association have been reported in many important crops, including pea (*Pisum sativum*), rice (*Oryza sativa*), and *Medicago truncatula* [95,96,97].

It is very clear that the plant–microbe interactions in the rhizosphere are influenced by several factors and our present knowledge is not sufficient to fully understand these complex interactions. As several studies have established that the rhizosphere microbiome composition greatly affects the plant health and, thus, the plant employs several mechanisms to recruit its specific microflora. Recent omics-based studies on next generation sequencing techniques are able to unravel the complex mechanisms employed by the plant to recruit its specific microflora, establishment of microbial communities in the rhizosphere, and finally its overall impact on plant health. This knowledge can further be utilized to increase crop quality and productivity in the changing climate scenario.

## 4. Impact of Organic Farming on Soil Biodiversity

Organic farming, mainly agriculture involving carbon-based amendments and various cover crops and avoiding the use of chemical fertilizers and pesticides, is a more sustainable practice in conservation agriculture. It is estimated that about 4.4 × 10^7^ ha of farm land is under organic agriculture across the world. Organic farming has been shown to positively influence the soil properties by improving the status of soil organic matter and soil nutrients with simultaneous reduction in soil erosion [21,22,23,24,25,26,27,28,29,30,31,32,33,34,35,36,37,38,39,40,41,42,43,44,45,46,47,48,49,50,51,52,53,54,55,56,57,58,59,60,61,62,63,64,65,66,67,68,69,70,71,72,73,74,75,76,77,78,79,80,81,82,83,84,85,86,87,88,89,90,91,92,93,94,95,96,97,98]. Microbial community structure, microbial biomass, soil carbon, and nitrogen are greatly influenced by changes in soil organic matter (SOM) and nitrate present in the soil. Hence, improvement in SOM and soil microbial activity (SMA) are better indicators for good soil health and quality under conservation agriculture [36,47,99]. In organic farming systems, the soil carbon is higher, which, in turn, provides more carbon-to-soil microbial community as a substrate that leads to changes in the soil microbial diversity [100]. Agricultural practices, such as MT, cover crops, and fertilization under conservation agriculture, are playing a vital role in microbial activity and biomass, leading to improvements in soil quality [36].

### 4.1. Cover Crops

Cover crop composition plays a key role in determining soil benefits, particularly when this is enriched with leguminous species, since they improve soil N status through fixation of nitrogen [36]. Cover crops especially hairy vetch (*Vicia villosa*) showed significant improvement in soil microbial biomass N (SMBN) and greater abundance of Gram-positive bacteria but lower abundance of mycorrhizal fungi than wheat and no-cover crops [36]. The higher abundance of actinomycetes and Gram-positive bacteria was attributed to the presence of higher aromatic carbon contents and anaerobic conditions in the soil [100]. Recently, cover crops have been used to control weeds in organic farming and the soil microbial communities have responded differently to different cover crop species. The effects of mixed species cover crop communities have been reported by Wortman et al. [101]. A total of 17 FAME biomarkers were influenced by cover crops in which 10, 5, and 2 FAME biomarkers were associated with bacterial, actinomycetes, and mycorrhizal functional groups, respectively. The abundance of these biomarkers was reduced significantly under weedy treatment as compared to cover crops [101].

### 4.2. Organic Amendments

Use of organic amendments is an important factor in organic farming where the crop productivity depends on the supply of soil nutrients, the production and composition of which is mediated through microbial decomposition of organic residues [102]. The type and amounts of organic substrate have been noted to have a significant influence on the abundance of the resident microbial community and its functional diversity [103,104]. Long-term application of organic wastes, such as animal waste, poultry litter, etc., has been reported to improve soil properties and increase diversity of bacterial community, particularly *Bacteroidetes*. Organic fertilization is reported to promote soil microbial diversity [28], while an increase in soil pH was observed along with an enrichment of *Acidobacteria* and depletion of *α-Proteobacteria* [105]. Dumontet et al. [106] compared the effects of different organic amendments on soil microbial metabolic activities. The results revealed that amendments with biochar resulted in greater diversity of cellulose-degrading bacteria.

## 5. Effect of Pesticides on Microbial Diversity

Commercial cultivation has enhanced the use of plant protection chemicals. Since they are active chemicals, they are bound to affect the soil microbiota strongly. Although some of the reports suggest that pesticides at their recommended doses have minor or transient effects [107,108] on soil microbiota in general, there is still a need of more specific studies as much as their effects on rhizospheric soil microbial structure as far as specific plant species are concerned. However, a clear description of the effects and side effects of pesticides on soil microbial diversity is also available [109,110]. Pesticides may have two kinds of effects on microbial diversity, the first being ‘immediate displacement’ of microbial communities due to pesticide toxicity, and the second, ‘long-term effects’ on microbial processes caused by succession of microbial communities. In both the cases, the microbial community structure shift may affect soil fertility [111]. Sun et al. [112], using multivariate regression tree analysis, reported that organochlorine pesticide levels are a second most important factor after the type of vegetation which, affects soil microbial diversity in pesticide contaminated soils. Diverse microbes play crucial roles in nutrient cycling and organic matter decomposition in soil [109]. A shift in the community composition due to external pressure (pesticides) may alter these activities. As we are aware of the fact that changes in land use from grassland to agriculture affects the community structure [113], use of agrochemicals could be one of the important factors behind it.

### 5.1. Microbial Metabolism of Pesticides

A few microbial groups use pesticides as a source of energy and nutrients, while others are affected by its toxicity. When a microbial community is affected, it disturbs the interwoven network of different trophic levels, leading to various indirect effects on soil microbial processes. Table 1, Table 2, Table 3 and Table 4 shows different chemical pesticides and their effects on microorganisms. For example, the herbicide sulphonylurea targets the synthesis of valine, leucine, and isoleucine, whereas, glyphosate stimulates C and N mineralization, indicating higher soil microbial activity and no effect on soil microbial biomass (Table 1) [114], but at higher doses, it reduces microbial biomass (3.84 L ha^−1^) [115]. Other herbicides, such as atrazine and paraquat, decrease dehydrogenase activity; however, paraquat persists for relatively longer times (up to 13 years) than atrazine (up to 100 days). The fungicide captan inhibits the activity of denitrifying bacteria, whereas fenpropimorph targets ergosterol biosynthesis as designed for leaf fungi (Table 2). Bjourland et al. [116], however, showed that this compound has no immediate toxic effects on bacteria, fungi, and protozoa of soil. The insecticide cypermethrin affects enzyme activities (namely, β-glucosidase, urease, acid-phosphatase, and dehydrogenase) [117] and has slight toxicity on soil biomass and other physiological activities for a short period (Table 3). On the other hand, the insecticide acetamiprid has a strong negative effect on soil respiration and was also found to affect phosphatase activity [118]. Soil fumigants also have deleterious effects on microbial activity (Table 4).

Some of the studies indicated that CO_2_ emission increases with pesticide application. This may be due to enhanced energy use to carry out cellular processes or due to the enhanced population of pesticide degrading microbial communities. In the latter case, the balance of diversity needs to be assessed. The rate of adaptation of microorganisms to pesticides may be considered important in maintaining equilibrium upon addition of agrochemicals, as there is an increase in the population of microbes able to degrade the agrochemical. Some of the microbial genes responsible for pesticide degradation have been identified, e.g., *lin*A and *lin*B genes have a role in the degradation of different forms of hydrocarbons along with their degradation intermediates [119,120,121].

**Table 1:** Different herbicides with their reported effects on soil microorganisms and biochemical reactions.

Herbicides	Effects on Microorganism and Associated Process	References
2,4-D	Adversely affects the activities of *Rhizobium* sp.	[122]
2,4-D	Reduces nitrogenase, phosphatase, and hydrogen photoproduction activities of purple non-sulfur bacteria.	[123]
2,4-D and 2,4,5-T	Adversely affects node-expression disrupting plant *Rhizobium* signaling. 2,4-D also reduces fixation by blue-green algae and nitrifying process impacting *Nitrosomonas* and *Nitrobacter* sp.	[124]
2,4-D, Agroxone, and Atranex	Inhibits activities of *Rhizobium phaseoli* and *Azotobacter vinelandii* (most sensitive).	[122]
2,4-D, Bromoxynil, and Methomyl	Reduces CH_4_ oxidation to CO_2_.	[125]
Bensulfuron methyl and Metsulfuron-methyl	Decreases N-mineralization.	[126]
Bentazone, Prometryn, Simazine, and Terbutryn	Inhibits N-fixation and decreases the number of nodules and N content overall.	[127]
Isoproturon, Triclopyr	Adversely impacts *Nitrosomonas*, *Nitrobacter*, urea hydrolyzing bacteria, nitrate reductase activity, and growth of actinomycetes and fungi.	[128]
Linuron, Terbutryn, and Methabenzthiazuron	Adversely impacts nitrogenase activity and nodulation at the pre-emergence application.	[129]
Glyphosate	Suppresses phosphatase activity.	[130]
Glyphosate	Reduces the growth and activity of *Azotobacter*.	[131]
Metribuzin	At lower doses, no effects on AM fungi in maize and barley.	[132]
Butachlor	Butachlor (20 μg/g) reduced the population of *Azospirillum* and anerobic nitrogen fixers in a non-flooded alluvial soil.	[133]
Metsulfuron-methyl, Chlorsulfuron, Thifensulfuron methyl	Reduced the growth of fluorescent psendomonads (77 strains).	[134]
Diuron, Linuron, Chlorotoluron	Negatively affect the microbial community structures.	[135]
Propanil, Prometryne	Propanil did not affect soil bacteria in general. Prometryne persisted in soil longer than propanil.	[136]
Glyphosate	Glyphosate produces a non-specific, short-term stimulation of bacteria at a high concentration.	[137]
Isoproturon	Affects the proliferation of *Sphingomonas* spp.	[138]
Butachlor	Negatively affects the general bacterial communities; the diversities ranged from 28% to 52%.	[139]
Diuron or Linuron	Removal of dominant acidobacterium.	[135]
Glyphosate	Increased relative abundance of β-Proteobacteria (*Burkholderia*).	[140]
Napropramide	Initial decrease in bacterial and fungal abundance followed by an increase in abundance of Gram-negative bacteria and fungi.	[141]
Pretilachlor	Decreased activity of phosphatase, urease, and dehydrogenase	[111]
Mesotrione	No response of the soil microbial communities in soil spread with field rate applications. Soil microbial activity stimulated by 100× FRA of pure Mesotrione.	[142]
Isoproturon	Treatment-induced changes in community composition	[109]
Imazetapir	Decreases nitrogenase activity in *Rhizobium leguminosarum*. *R. trifolii, Bradyrhizobium* sp., and *Sinorhizobium meliloti*.	[143]

**Table 2:** Different fungicides with their reported effects on soil microorganisms and biochemical reactions.

Fungicides	Effects on Microorganism and Associated Process	References
Fenpropimorph	Fenpropimorph inhabited the growth of active fungi and calculable bacteria.	[144]
Iprodione	Affects the soil bacterial communities.	[145]
Apron, Arrest, and Captan	Reduces viable counts of *Rhizobium cicero*.	[146]
Benomyl	Impacts mycorrhizal associations and nitrifying bacteria.	[147]
Benomyl, Mancozeb	Arrests activity of dehydrogenase, urease, and phosphatase.	[148]
Captan	Inhibits aerobic N-fixing, nitrifying, denitrifying bacteria, nitrogenase activity, phosphate solubilization, and other fungi.	[149]
Captan and Thiram	Decreases cell growth and nitrogenase activity in *Azospirillum brasilense*.	[150]
Captan and Carbendazim	Decreases the activity of nitrogenase enzyme.	[123]
Captan, Carboxin, Thiram	Inhibits the activity of bacteria responsible for denitrification.	[151]
Carbendazin and Thiram	Inhibits nodulation in legumes and thus N-fixation process.	[143]
Chlorothalonil	Affects bacteria associated with nitrogen cycling.	[147]
Chlorothalonil, Azoxystrobin	Affects biocontrol agent(s) used against Fusarium wilt.	[152]
Copper fungicides	Decreases population of bacteria, cellulolytic fungi, and *Streptomycetes*.	[153]
Dimethomorph	Inhibits nitrification and ammonification process.	[154]
Dinocap	Inhibits the activity of ammonifying bacteria.	[155]
Dithianon	Destroys bacterial diversity.	[156]
Fenpropimorph	Slows down bacterial activity.	[151]
Fludioxonil	Toxic to algal activities.	[157]
Funaben, Baytan, Oxafun	Inhibits nitrogenase activity of methylotrophic bacteria.	[158]
Hexaconazole	Impacts bacteria involved in N cycling.	[159]
Mancozeb	Impacts on bacteria involved in the N & C cycle.	[155]
Mancozeb, Chlorothalonil, Metal dithiocarbamates	Reduces nitrification process.	[160]
Metalaxyl	Reduces urease activity continuously while phosphatase activity seems stimulated but then reduces.	[161]
Metalaxyl	Disturbs activity of ammonifying and nitrifying bacteria.	[162]
Oxytetracycline	Acts as bactericide.	[163]
Pencycuron	Short-term impact on metabolically active soil bacteria.	[164]
Propiconazole	Retards PGP effects of *Azospirillum brasilense* on its host plant.	[165]
Triadimefon	Deleterious to long-term soil bacterial community.	[166]
Triarimol and Captan	Reduces frequency of *Aspergillus* sp.	[167]
Azoxystrobin, Chlorothalonil, Tebuconazole	None of the fungicides affected bacterial community structure. Chlorothalonil negatively affect the ciliate protozoan *Arcuospathidium* sp., or *Bresslaua vorax*. Azoxystrobin affect the Flagellate protozoan *Paraflabellula hoguae*, while ascomycete fungus *Cladosporium tenuissimum* was affected by tebuconazole.	[162]
Cobber	Bioavailable Cu positively correlated with relative abundances of phylums *Acidobacteria* and negatively correlated with the phylums Proteobacteria and Bacteroidetes.	[168]
Cobber	Decrease in abundance of acidobacteria and increase of Firmicutes. *Bacillus* community highly resistant to high cobber concentrations.	[169]
Mancozeb	Enhanced activity of alkaline phosphatase, protease, amidase. Decreased activity of urease and asparaginase	[170]
Propiconazole	Decreased activity of phosphatase, urease, and dehydrogenase.	[111]
Chlorothalonil	More transient and weaker negative effects on soil micro-organisms.	[171]
Thiram	Diversity decrease at 200 mg kg^−1^.	[172]
Tebuconazole, Metalaxyl	Perturbation of bacterial community structure compared to control.	[173]
Carbendazim, Thiram	Decreases nitrogenase activity in *Rhizobium leguminosarum*. *R. trifolii, Bradyrhizobium* sp., and *Sinorhizobium meliloti*.	[143]
Metalaxyl and Mefenoxam	Decreases nitrogen-fixing bacteria and microbial biomass.	[174]

**Table 3:** Different insecticides with their reported effects on soil microorganisms and biochemical reactions.

Insecticides	Effects on Microorganism and Associated Process	References
Cypermethrin	Increase in Gram-negative bacteria and decrease in firmicutes.	[175]
Amitraz, Aztec, Cyfluthrin, Imidachlorpid, and Tebupirimphos	Reduces activities of urease and phosphatase enzymes.	[176]
Arsenic, DDT, and Lindane	Decreases microbial biomass and microbial and enzymatic activities.	[177]
Bensulfuron methyl and Metsulfuron-methyl	Reduces soil microbial biomass.	[178]
Carbamate	Inhibits several soil microorganisms, enzymes, and nitrogenase activity of *Azospirillum*.	[130,179]
Carbofuran, Ethion	Inhibits nitrogenase activity of *Anabaena doliolum*.	[180]
Chlorinated hydrocarbons	Inhibits methanogenesis.	[181]
Chlorpyrifos, Dichlorvos, Phorate, Monocrotophos, Methyl parathion, Cypermethrin, Fenvalerate, Methomyl and Quinalphos	Increases phosphatase activity initially and later reduces gradually. Phorate reduces the total bacterial population and N-fixing bacteria.	[182]
Chlorpyrifos, Profenofos, Pyrethrins, and Methylpyrimifos	Reduces the population of aerobic N-fixing, nitrifying and denitrifying bacteria, and several fungi. Profenofos and Pyrethrins decrease the activity of urease enzyme and nitrate reductase.	[183]
Chlorpyrifos, Quinalphos	Reduces the ammonification process.	[182]
Cyfluthrin, Fenpropimorph, and Imidacloprid	Decreases the nitrification and denitrification process. Stimulates sulfur oxidation.	[176]
Diazinon and Imidacloprid	Inhibits a urease-producing bacterium (*Proteus vulgaris*).	[184]
Lindane, Malathion, Diazinon, and Imidacloprid	Lindane inhibit state of nitrification, N-availability, P-solubilization, and activity of phosphomonoesterase enzyme, while the opposite effect is observed in the case of Diazinon and Imidacloprid.	[177]
Methamidophos	Reduces microbial biomass by 41–83%.	[185]
Neemix-4E	Reduces urease enzyme activity.	[186]
Organophosphate insecticide	Impacts the activity of soil enzymes, several beneficial soil bacteria, and fungal population and reduces N-mineralization rate.	[179]
Pentachlorophenol	Reduces nitrification.	[187]
Quinalphos	Reduces activity of phosphomonoesterase.	[188]
Diflubenzuron	Diflubenzuron (100–500 μg/g) stimulates dinitrogen-fixing bacteria (*Azotobacter vinelandii*).	[189]
Methylpyrimifos, Chlorpyrifos	Methylpyrimifos (100–300 μg/g) or chlorpyrifos (10–300 μg/g) significantly decreased aerobic dinitrogen-fixing bacteria. Fungal populations and denitrifying bacteria were not affected.	[190]
Fenamiphos	Not toxic to dehydrogenase or urease activities, but likely to be detrimental to the nitrification in the soil.	[191]
Methamidophos	High concentrations of methamidophos (250 mg/kg) stimulate fungal populations. DGGE fingerprinting patterns showed a significant difference between the responses of culturable and total fungi communities under the stress of methamidophos.	[192]
Methamidophos	Methamidophos at 0.031 g/pot/week and 0.31 g/pot/week significantly decreases microbial biomass by 41–83% compared with the control.	[185]
Methylparathion	Induced the community of γ-porteobacteria (*Pseudomonas stutzeri* and *Pseudomonas putida*).	[193]
Carbaryl, Carbofuran	Carbary (10 μg/g) had almost no effect on nitrogenise; however, carbofuran (2 μg/g) reduced the population of *Azospirillum* and anerobic nitrogen fixers. Carbofuran (4 μg/g) stimulated the population of *Azospirillum* and other anaerobic nitrogen fixers.	[133]
Profenofos	Decreased activity of phosphatase, urease, and dehydrogenase	[189]
	Higher activities at lower dosage, greater toxic effects at higher dosage.	[194]

**Table 4:** Different soil fumigants with their reported effects on soil microorganisms and biochemical reactions.

Soil Fumigants	Effects on Microorganism and Associated Process	References
Metam sodium	Dose-dependent shift in community structure (after 5 weeks).	[195]
Methyl Bromide	Increased abundance of Gram-positive bacteria.	[196]
Methyl isothiocyanate	Increased abundance of Gram-positive bacteria.	[196]
Metam sodium	Inhibitory effect on Gram-negative bacteria and fungi in both field and laboratory studies.	[197]
1,3-dichloropropene	Initial inhibition of dehydrogenase activity (at 500 mg kg^−1^). Bacterial community diversity decreased with higher concentration.	[126]

### 5.2. Deleterious Effects on Microbial Community

Prior to granting approval to a pesticide compound, its effects on microbial processes are assessed [198] by measuring microbial activities in soil [199]. The effect of pesticides on carbon and nitrogen metabolism after adding organic substrates to the soil was also assessed. The transformation of compounds such as nitrate, nitrite, ammonium, oxygen, and carbon dioxide are used to study the effects of pesticide on soil environment, the deleterious effects of which must be below 25% for approval in the United States of America [199]. However, even a great shift in bacterial community structure may not always result in any significant change in overall nitrogen and carbon metabolism. Some other species which could metabolize the pesticides pre-dominate the scenario, suppressing those which are sensitive to the effects of the pesticide in question. The effects of pesticides on the overall dynamics of microbial diversity can be influenced by the fact that some microbial communities may be able to use the pesticide as a source of energy and nutrients, while others are affected by its toxic effects. Metagenomic-based studies indicated abundance of the bacterial genera *Pseudomonas*, *Sphingomonas*, *Novosphingobium*, *Sphingopyxis, Marinobacter*, *Chromohalobacter*, *Halomonas*, and *Alcanivorus* at a dumpsite of hexachlorocyclohexane (HCH) [119]. Johnsen et al. [111] discusses the consequences that the shift in microbial community structure experiences due to a vacant ecological niche created by suppression of one microbial community, and how this succession leads to altered ecological activities in due course of time. Therefore, it is important that the effects of pesticides on microbial diversity at different levels should be assessed in both immediate and long-term studies. Metagenomic studies in this respect could be more useful.

Several ecological indicators have been worked out to assess the impact of pesticides on soil microbial activity. They range from assessing microbes as a whole to soil microbial biomass [200], enzyme activity [170,201], mineralization rate [202,203], community-based profiling (physiological profiling) [204], DNA based profiling [205], and fatty acid-based profiling [206] (to assess the community shift), and meta-omic approaches [119]. All these techniques warrant different degrees of accuracy and relationship with soil biogeochemical processes. Meta-omic approaches were able to decipher the phenomenon at the level of genes responsible for degradation of target pesticides [207,208,209] which could further give clues to expedite the process of residue clean-up from agricultural lands.

### 5.3. Methods of Detecting Effects of Pesticides on Microbial Community Structure

The methods and basis used for detecting effects of pesticides on microbial diversity are of paramount importance, because what is going to be measured should be a true indicator of what is actually happening in the ecosystem. In an aquatic ecosystem, Widenfalk et al. [109] reported that community level end points (measuring microbial activity and biomass) did not become affected by pesticide exposure, but on the other hand, subcommunity level endpoints (16s rRNA-based genotyping, T-RFLP, and PLFA composition) were affected by pesticide exposure, thus being better indicators to detect the changes in the resident microbial community caused by pesticides. One of the reasons for observing no effects on community level end points is compensatory mechanisms. In lower doses of pesticides, the microbial activity becomes affected but only with higher doses is the inhibition of bacterial activity observed. Widenfalk et al. [210] suggested that some microbial groups were favored by high pesticide exposure and masked the overall impact on microbial activity and biomass. Similar microbial community shifts in soil have been reported by El fantruossi et al. [135]. Following pesticide application, some of the microbial communities capable of degrading pesticides [211] are increased in number in the total microbial population. For subcommunity level end points, traditional culture-based diversity studies could not reveal a complete picture of change in populations as they represent only less than a percent of total soil microbiota; therefore, meta-omics-based studies could provide a more complete understanding of the change in diversity of the resident microbiota.

In a more conclusive way, gene expression as affected by pesticide application was studied by several workers. Expression of the *amoA* gene, which is involved in ammonia oxidation, was found to be decreased in a soil microcosm exposed to dazomet and mancozeb using reverse-transcription qPCR. Additionally, bacterial diversity analysis using 16S ribosomal RNA sequencing is also affected by pesticides. Long-term inhibition was observed in bacterial and archeal *amoA* transcript numbers by two log units for more than 28 days by dazomet, whereas mancozeb inhibited *amoA* transcripts transiently. The inhibition of total bacterial numbers by one log unit was observed in 12 days by dazomet but was later restored. However, firmicutes and proteobacteria were dominating classes on day twelve, indicating a halt in early opportunists’ growth and the initiation of re-establishment of a diverse population. On the other hand, no effect of mancozeb on bacterial diversity was observed.

## 6. Effect of Moisture Levels on Soil Microbial Biodiversity

Microbial diversity of soil is an important soil health index. Ecosystem biodiversity is positively related to its resilience and stability [61,212]. Any harm to the ecosystem negatively impacts its biodiversity [61,213]. Soil moisture content is one of the most important factors that affect the microbial diversity, as it affects the availability of free water connecting soil particles, which are crucial for microbial life. Zhou et al. [214] found that the bacterial diversities are reduced in water-saturated soil. Complete flooding eventually leads to anoxic conditions and has a significant impact not only on soil properties but also on the complete soil ecosystem [215]. According to Denef et al. [216], anoxic conditions created by alternating wetting and draining of the soil disturbed the normal soil microbial population structure by favoring or suppressing the growth of particular microbial communities. Wetting and drying state cycling negatively influences the population of obligate aerobes and anaerobes, but will support the growth of microorganisms tolerant to both the conditions. Soil microbial population is also influenced by the flood duration as a result of a decrease in the rate of change in redox potential in anaerobic conditions [217], and thus, the rate of denitrification, reduction of iron and sulfur, and methanogenesis [218] might be affected. During the dry period, nitrification and denitrification rates slow down but resume after rewetting of soil [219]. Conrad [220] reported that anaerobic soils may contain methane producing microorganism, especially Archaea, which under strict anaerobic conditions produce methane gas (having a high global warming potential). Short-term drainage of floods in rice field significantly reduced methane emission [221,222]. This was expected as methanogens can only thrive in very low levels of oxygen [220].

Some facultative anaerobic bacteria, such as *Methylosinus trichosporium* and *Mycobacterium smegmatis* could survive under temporary hypoxic conditions [223]. Growth of these microbes is negatively influenced by frequent wetting and drying. However, radical changes in the community dynamics of soil bacteria were observed when dry lands were transformed into agricultural land [224]. Conversion from hyper-arid deserts to agricultural land resulted in an increased bacterial diversity [224]. Since soil water content plays a crucial role in regulating oxygen diffusion, the moisture levels between 50–70% of water-holding capacity exhibit maximum aerobic microbial activity [100,225], and thus, can be considered as optimum for normal microbial growth and development. Excess moisture levels also decrease the rates of organic matter decomposition due to restricted oxygen availability. On the other hand, low soil moisture content results in reduced microbial and soluble substrates mobility along with cellular water potential, thus affecting activities of the soil microflora negatively [214,226]. Geyer et al. [227] indicated that in polar desert soils the population of several bacterial genera was significantly dependent on soil moisture levels.

Bacterial communities are rather stable with a change in the soil moisture content as compared to fungal communities [228]. Among the bacteria, the population of Proteobacteria is significantly affected by a change in the soil moisture content [229]. Along with the moisture level, soil temperature also influences microbe–microbe interaction and diversity as a whole. This signifies the role of soil nutrient and water use efficiency for a healthy population structure of microbes in the soil [230]. The soil moisture content also affects rhizosphere colonization [231] as it is crucial for the mobility of bacteria. Bachar et al. [232] reported that precipitation has more significant effects on abundance of bacteria rather than on its diversity. Decreased soil moisture content as a result of global warming will likely limit survival, dispersal, and colonization ability of microorganisms in soil spaces [233], and thus, it might have a role in modification of the rhizosphere microbiome structure. Therefore, modulation in the soil moisture content along with other agricultural practices could enhance the soil microbial diversity, which can be utilized further for sustainable crop production in the changing environmental scenario.

## 7. Microbial Inputs: A Way Out for Sustainable Crop Production

An active and diverse soil biota is important for maintaining crop productivity and quality, and preservation of these traits is a major goal of sustainable farming. Agriculture is an age-old practice, being followed over thousands of years and never leaving any kind of ill effects on soil, human health, and even on the environment until comparatively very recently, after the introduction of inorganic agrochemicals. Agriculture, being the main source of income and employment in the country, is rightly considered the backbone of the Indian economy. Within the last fifty to sixty years, as a result of the adoption of many faulty agricultural practices, the sustainability of the entire agricultural industry has become debatable. India is an agriculture-based country where more than 50% of its population depends on an agricultural economy. Toxic or ill effects of agrochemicals incorporated in soil knowingly or unknowingly in the course of various crop management practices can be remediated through application of potentially beneficial soil microorganisms. Soil microorganisms having different agriculturally important traits may be used for different agriculturally beneficial activities, i.e., bioremediation, nutrient cycling, nutrient acquisition, making suppressive soils, biological control, etc.

Agricultural intensification may alter soil biodiversity in a manner that affects the overall ecosystem function. Soil microbial communities are strongly affected by different agricultural practices, especially the application of organic amendments [234]. To maintain healthy environments, new technologies need to be applied, including microbial inoculations into the soils. There are a number of approaches, which can be used, on a sustainable basis, to meet food requirements without compromising environmental health. Among these, the use of microbial products is pivotal to ensuring food security in a changing climate [235]. The fact that microbial approaches can successfully be used for sustainable agricultural development has well been established and proven by a number of examples. Several microbial formulations provide alternatives to the two most important categories of toxic agrochemicals/soil pollutants, namely, inorganic fertilizers and chemical pesticides, are available on the market which are being used effectively by farming communities to enhance crop production without any ill effects on local natural resources including soil, water, and even on the environment. Rhizosphere engineering through the manipulation of effective microorganisms and agricultural management practices, such as cropping pattern adaptation, intercultural applications, irrigation scheduling, crop geometry, etc., may be other potential alternatives for bringing sustainability in agriculture [234,235].

Farmers are advocated and forced to apply advanced and new agricultural technologies to increase production/yield, and in this process, they are likely to apply more agrochemical-based inorganic fertilizers and chemical pesticides. This trend is predominately seen in the agriculture policies of developing countries. Industrialization and other anthropogenic activities, especially those considered essential by developing countries, result in the production of pollutants, which accumulate continuously over time in soils dedicated to agricultural use, and also in aquatic environments and contaminating them likewise. Plants are entirely dependent upon native microorganisms to utilize soils as a growth medium, and the synergy between both is important for their survival. The main challenge in current agricultural research is to meet sustainable environmental and economic issues without compromising yields and produce quality. Looking at the present context, exploiting the agroecosystem services of soil microbial communities appears a promisingly effective approach to meet this challenge. Nowadays, emphases are being given to develop green technologies that can degrade toxic pollutants already incorporated into nature in order to bring their levels down to what we consider “safe”. One suitable eco-friendly alternative approach is exploiting the role of soil microbial communities for sustainable and healthy crop production, while preserving the biosphere. Indeed, soil microorganisms play fundamental roles in agriculture by being pivotal to a number of processes that may lead to various direct and indirect beneficial effects for crop plants, i.e., crop-residue decomposition and nutrient cycling, improving plant nutrition and health, as well as soil quality as substrates for plant growth. Hence, several strategies for more effective exploitation of beneficial microbial services, well-recognized low-input biotechnology to help sustain environmentally friendly agrotechnological practices have been, and are being, advocated. These recommended agricultural practices aim to optimize the role of root-associated microbiome in crop production by nutrient supply and plant protection exploiting biological activities. Since the interactions between microbial communities and crops are influenced by diverse ecological factors and agronomic management, the impact of environmental stress factors with various crop-microbe interactions needs to be considered, particularly in the current global climate change scenario. Diverse types of stress situations are generated by intensive agricultural practices, and all of them are affecting the functionality/productivity of both agricultural systems and natural ecosystems, and therefore, restrict various ecosystem services. A number of stress factors still prevail and these include salinity, drought, nutrient deficits, contamination, soil erosion, diseases, pests, invasive plants, etc. Besides creating hazards, agrochemical application for crop production and protection provokes environmental contamination and may still pose threats to human health. Most of these microbes remain in the rhizospheric soil or rhizo-plane, but a small subpopulation of them, designated as “endophytes”, are able to penetrate and live within plant tissues. These little friends of agriculture also have several beneficial effects on plant growth and its overall performance without showing their presence. Endophytes are known to have plant growth promoting, nutrient fortifying, and biotic and abiotic stress alleviating potential for different crop plants and have greater potential to be used as microbial inoculants [236,237,238,239]. In a similar way, plant growth promoting rhizobacteria is reported to influence plant growth and protect plants from various biotic and abiotic stresses along with biofertilization and biofortification in crops of nutritional importance [240,241,242].

## 8. Conclusions and Future Prospects

Increasing and diversifying global food demands are mounting pressures on agricultural production, and hence, are becoming major challenges to contemporary agriculture. To meet the food requirement of a burgeoning population, especially in developing countries, agricultural intensification has become inevitable. The surging demand for food can justifiably be fulfilled only through increased crop production, while utilizing available resources in a sustainable way. The increasing rate of urbanization coupled with rising income and changing dietary patterns in the wake of growing health awareness among the population are leading to an increase in demand for different types and varieties of food. The need for a high-quality diet and the rising popularity of organic produce are going to require additional resources for crop production in the days to come. All these factors eventually put significant pressure on the agricultural system, in general, and on microbial diversity, in particular. Now, as we realize the need of ensuring long-term sustainability with optimal resource use efficiency and that too without negative effects in the restricted land whatsoever available for agricultural cultivation, a variety of management practices are being developed. Manipulating the rhizosphere with desirable changes in soil microbial diversity could improve plant performance by influencing water dynamics and enzyme activities. Agricultural management practices that promote soil organic matter (SOM) accumulation and retention enhance microbial biodiversity of soil in general and the plant rhizosphere in particular. To maintain a healthy soil environment, new technologies need to be applied, including microbial inoculations and measures to ensure their retention in the system for the desired periods. There are a number of approaches which can be used on a sustainable basis to meet food requirements without compromising environmental health. Among these, use of microbial products is becoming pivotal to ensuring food security in a changing climate. Rhizosphere engineering through manipulation of effective microorganisms and agricultural management practices, such as crops and cropping pattern adaptation, intercultural applications, irrigation scheduling, crop geometry, etc., may be the alternatives that can be integrated suitably for system sustainability. Looking into the present context, exploiting the agroecosystem services of soil microbial communities appears to be a promisingly effective approach to agriculture in the days to come. One of the suitable and eco-friendly alternative approaches is exploiting the role of soil microbial communities for sustainable and healthy crop production. Therefore, several strategies for more effective exploitation of beneficial microbial services and low-input biotechnology may be coupled to develop environmentally friendly agrotechnological practices. This can be propagated and advocated among large farming communities. Potential of endophytes can also be explored for restoring soil system sustainability. Despite considerable advancements in DNA sequencing technologies, the knowledge of the effect of different tillage practices on taxonomy and phylogenetic composition of microbial communities is still limited. The effects of different tillage practices under various soil types and climatic conditions on soil microbial diversity need to be studied in detail for better understanding of the system. In a nutshell, it can be concluded that although there have been remarkable achievements in agriculture with the application of microbial biotechnology, opportunities still need to be explored based on specific agricultural practices and soil microbial interactions for sustainable agricultural development in the future. This knowledge based on specific agricultural practices and soil microbiology can be exploited for the identification of indicators not for soil health only but also for agricultural health in toto. There is an urgent need of emphasizing the adoption of newer techniques in agriculture which may ensure ecosystem sustainability while simultaneously maintaining food security and quality for the ever-increasing population.
